# Chicago Society of Physicians and Surgeons

**Published:** 1873-08-15

**Authors:** 


### Society Reports.

### CHICAGO SOCIETY OF PHYSICIANS AND SURGEONS.

The regular meeting of the Society was held on Monday evening, July 28th, President Dr. A. Fisher in the chair.

Dr. F. H. Davis read a report of two cases of Sporadic Cholera, and Dr. Ethridge followed with an extended and interesting report on the Pathology and Treatment of Cholera. Dr. Ethridge considered that an overstimulated, exalted condition of the vaso-motor nervous system constituted the essential pathological conditions present in cholera, and proceeded to show how the various symptoms and phenomena of the disease could be explained on this theory. In regard to treatment, he thought that, admitting the correctness of this theory, such remedies as exerted a quieting, sedative influence upon the vaso-motor nervous system, ought to act beneficially. Having had no experience himself in the treatment of epidemic cholera, he had interviewed some dozen or more of what he called representative physicians, for the purpose of obtaining their opinion regarding the most successful courses of treatment. These opinions, however, as detailed by the Dr., were chiefly remarkable for the variety and diversity of the views expressed, no two out of the number, apparently, agreeing fully in their ideas regarding the pathology or treatment of the disease.

At the close of Dr. Ethridge's paper, Dr. John Bartlett made a few remarks regarding his experiences while in charge of the Cholera Hospital at Louisville, in 1866. The majority of the patients were in collapse when they arrived, so that the percentage of deaths in the Hospital had been necessarily very large. Almost everything in the way of treatment that was, or ever had been, suggested, was given a trial by them at that time—teaspoonful doses of calomel, repeated every half hour; the injection of artificial serum into the veins, and such like heroic measures among the rest. The Dr. mentioned one expedient which he had sometimes resorted to to arrest vomiting, and which he said might be denominated *splinting the stomach.* An ordinary washbowl being inverted over the abdomen, with a bit of lighted candle underneath to exhaust the air, a large *dry-cup* was thus formed, which, he stated, would in almost every instance effectually prevent any further effort at vomiting. In fact, he had never seen but one or two persons who *could* vomit, with this splint effectually applied.

In closing, Dr. Bartlett said, that, without any reference to the so much disputed question of pathology, there *were some remedies* which *experience* had demonstrated were useful, and which, when resorted to in time, were generally successful in arresting the attack of cholera. Chief among these were calomel, morphine, and acetate of lead, in small doses, frequently repeated. In the stage of collapse, strychnia and atropia had also sometimes proved of value. In giving injections in cholera, he said that care should be taken to introduce the injection well up into the rectum, by means of a small tube or canulæ fitted to the nozzle of the syringe.

At the request of the Society, Dr. Simons made a few remarks regarding some cases of cholera of a malignant type that had fallen under his care, on Butterfield street, near Thirty-ninth street. A full consideration of these cases was, however, postponed until the next meeting, when Drs. Boyd and Simons would be prepared to give a more detailed account of the epidemic which had apparently started in that region.

The Society then adjourned.

### CHICAGO SOCIETY OF PHYSICIANS AND SURGEONS

#### REGULAR MEETING.

Monday evening, Aug. 11th.—The Society was called to order by the President, Dr. Fisher. On recommendation of the Board of Censors, Drs. Lester Curtis and I. N. Danforth were elected members. On motion, the report of the Section on Surgery, which would have been the regular business to come before the Society at the meeting, was postponed for two weeks, in order to allow of the presentation of the reports on Cholera, prepared, at the request of the Society, by Drs. Boyd and Wood.

Dr. W. H. Boyd presented a map of localities where cases of cholera had occurred under his notice. He first cited the case of a man living at No. 945 Butterfield street. He was taken at about 4 o'clock A.M., and, when seen by Dr. Boyd, was in a state of collapse. He died in about six hours, and was buried the next day by his son, who rode to the grave on the corpse. The son was taken at 4 o'clock P.M. and died at 11 in the evening; and the mother died of the same complaint the following day. Dr. Boyd also referred to the case of a brakeman, who had not gone into any of the infected houses, nor had any intercourse with any of the families attacked by the disease, but who had passed one of the houses several times, and had undoubtedly taken it from the discharges thrown out promiscuously in the streets and gutters.

Dr. Jackson also knew of cases where the discharges had been thrown out and left exposed. An emigrant family moved into the house and three of them died.

In continuing the lecture, Dr. Boyd showed a well-defined connection between all the cases attended by him since June 9, amounting to 70 in all, and the first cases occurring at No. 945 Butterfield street. Dr. Boyd stated that there had been, in all, 36 deaths in this district from the disease, of which 17 were his cases. Wherever he had been called early, nearly all recovered; but when he had not been called until the state of collapse had ensued, only about five per cent. recovered.

The localities embraced in Dr. Boyd's practice are bounded by Thirty-seventh, Burnside, Forty-second streets, and Wentworth avenue, about 25 blocks in all.

Dr. M. W. Wood read an extended and important paper upon the various symptoms and treatments in cholera cases, and gave a very thorough citation of authorities. He expressed the opinion that cholera was the result of a specific malarial poison, assisted by local causes.

Dr. J. R. Jackson stated that from all the symptoms described there seemed to be but little difference between these symptoms and those of genuine cholera; and it was an important point to determine whether these cases should be acknowledged as cholera, or whether, as demanded by the Board of Health, they should continue to call it cholera-morbus. Dr. Rauch had gone over the territory with Dr. Boyd, and had attributed the prevalence of this disease to the character of the water, and recommended that the water service be extended to this locality. This had been done, but without marked improvement. It is now a question whether the Society should call this disease cholera, cholera-morbus, cholerine, or choleraic diarrhœa. If it was cholera, let them say so. The Board of Health seem to desire to keep the matter secret, for fear of creating an excitement. The disease should be stopped in the outset in the locality where it originated.

Dr. Simons said that the Board of Health objected to the disease being reported as cholera on account of the demoralizing effect of such an announcement. He thought it was cholera, and contagious. Dr. Miller had made arrangements to disinfect every place where any case was reported. The sanitary police had orders to report every case immediately, and to remove individuals from crowded houses to the hospital. Proper nurses and attendants would be provided for indigent families. Diarrhœa almost always was a premonitory symptom, and when it occurs medical aid should be called immediately.

Dr. Ethridge said that, since 7 o'clock this evening he had attended three cases on Butterfield street, near Thirty-second, a woman, son and daughter. Symptoms were all similar to cholera.

Dr. Jackson wished to know the opinion of the Society upon the actual sanitary condition of the city.

Dr. Lyman thought that we had cholera in a sporadic form, but not Asiatic cholera.

Dr Emmons thought it was Asiatic cholera in the sporadic form. The city was not in a good condition. He advised caution in the matter of diet, and avoidance of food which tended toward cholera.

Dr. Hyde advocated the erection of pavilion hospitals in isolated places, which could be entirely destroyed after the disease had passed by.

Dr. Simons said that the Board of Health had fitted up the old small-pox hospital, and also intended to ask Gen. Sheridan for a number of shelter-tents, to be used in the open spots.

Dr. Bartlett thought that the disease was cholera Asiatica, and nothing else. He wanted to know what the Board of Health had been doing, and what they were paid for, if they permitted such a state of affairs as had been spoken of by Drs. Boyd and Jackson. The condition of many parts of the city was outrageous.

After a prolonged discussion the Society adjourned, on motion, for one week, when a special meeting will be held for the further discussion of the subject of Cholera.

